# The current status of ethnobiological research in Latin America: gaps and perspectives

**DOI:** 10.1186/1746-4269-9-72

**Published:** 2013-10-16

**Authors:** Ulysses Paulino Albuquerque, Josivan Soares Silva, Juliana Loureiro Almeida Campos, Rosemary Silva Sousa, Taline Cristina Silva, Rômulo Romeu Nóbrega Alves

**Affiliations:** 1 Universidade Federal Rural de Pernambuco, Laboratório de Etnobiologia Aplicada e Teórica (LEA), Rua Dom Manoel de Medeiros, s/n, Dois Irmãos, Recife/PE CEP: 52171-900, Brazil; 2 Departamento de Biologia, Universidade Estadual da Paraíba, Av. das Baraúnas, 351/Campus Universitário, Bodocongó, Campina Grande-PB 58109-753, Brazil

**Keywords:** Ethnobotany, Ethnomedicine, Ethnopharmacology, Ethnoveterinary, Ethnozoology, Scientometrics

## Abstract

**Background:**

Recent reviews have demonstrated an increase in the number of papers on ethnobiology in Latin America. Among factors that have influenced this increase are the biological and cultural diversity of these countries and the general scientific situation in some countries. This study aims to assess the panorama of ethnobiological research in Latin America by analyzing its evolution, trends, and future prospects.

**Methods:**

To conduct this study, we searched for papers in the Scopus (http://www.scopus.com) and Web of Science (http://www.isiknowledge.com) databases. The search was performed using combinations of keywords and the name of each Latin American country. The following countries were included in this study: Argentina, Bolivia, Brazil, Chile, Colombia, Costa Rica, Cuba, Ecuador, Guatemala, Haiti, Honduras, Mexico, Panama, Paraguay, Peru, Venezuela, and Uruguay.

**Results and conclusions:**

According to our inclusion criteria, 679 ethnobiological studies conducted in Latin America were found for the period between 1963 and 2012. Of these studies, 289 (41%) were conducted in Brazil, 153 in Mexico (22%), 61 in Peru (9%), 58 in Argentina (8%), 45 in Bolivia (6%), and 97 (14%) in other Latin American countries. The increased number of publications related to this area of knowledge in recent years demonstrates the remarkable growth of ethnobiology as a science. Ethnobiological research may be stimulated by an increase in the number of scientific events and journals for study dissemination and by the creation of undergraduate courses and graduate programs to train ethnoscientists who will produce high-quality studies, especially in certain countries.

## Introduction

Throughout history, people have interacted with their environment in multiple ways. These direct and indirect dependent interactions with natural resources have resulted in historical relationships that are extremely important to human societies [1], evidenced, for example, through the utilization of natural resources for subsistence and commercial purposes [2]. Such interactions can be studied from an ethnobiological perspective. As a science, ethnobiology, understood by Posey [3] as a “study of knowledge and concepts developed by any society about the plant and animal world, encompassing both the way in which a social group classifies plants and animals, and the use that gives them”, investigates the complex past and current relationships between people and the environment.

The oldest records and contributions to ethnobiology were made by the naturalists of the Old World (Egyptians, Greeks, and Romans) and explorers of the New World (Europe). In addition to unveiling new lands, these records speculated about the use of species by native people [4]. This moment is known as Phase I (pre-classical period) of ethnobiology (the first half of the 19th century until 1950), in which works were descriptive and utilitarian, documenting the use of plants and animals through lists of species [4-6]. Studies in the classical period (1950 to 1980) include analyses related to linguistics and ethnobiological classification as well as the publications of studies of the management of natural resources by different ethnic groups [4]. These studies promoted the interaction of ethnobiology and conservation, which has been the focus of ethnobiological studies during the post-classical period (1981 to the present) [4].

The importance of ethnobiological studies has been recognized, especially among conservation biologists, only in the last 20 years, primarily due to increased appreciation of the strong human influence on biodiversity (see [5,7,8]). Ethnobiological studies are currently being conducted not only by anthropologists (who were pioneers in the area) but also by researchers in other fields, such as botany, zoology, ecology, and agronomy. The involvement of these researchers reflects the academic growth in the field of ethnobiology and its multidisciplinary character. The multidisciplinary characteristics of this science allow a broad spectrum of approaches and applications as well as the appearance of various areas of knowledge related to ethnobiology, such as ethnobotany, ethnozoology, ethnoecology, ethnomedicine, and ethnopharmacology. Recent reviews have demonstrated a notable increase in the number of publications on ethnobiology in Latin America (see [5,9-11]), mainly in Brazil, Colombia, and Mexico. These studies have had a significant impact on scientific production in Latin America. However, an integrated analysis of the ethnobiological studies produced in these countries is lacking. Therefore, this study aims to assess the panorama of ethnobiological research in Latin America by analyzing its evolution, trends, and future prospects using a broad concept of ethnobiology that covers all the sciences listed above, although there are different views of this concept. This study is based on the following questions: How many studies have been published per country and per area of knowledge? How has the number of publications varied over time in different areas of knowledge and in different countries? Does any variation exist in the numbers of citations and international collaborations with regard to the number of publications per country or area of knowledge?

## Methods

To conduct this study, we searched for papers in the Scopus (http://www.scopus.com) and Web of Science (http://www.isiknowledge.com) databases. The search was performed using combinations of keywords and the name of each Latin American country. The following countries were included in this study: Argentina, Bolivia, Brazil, Chile, Colombia, Costa Rica, Cuba, Ecuador, Guatemala, Haiti, Honduras, Mexico, Panama, Paraguay, Peru, Venezuela, and Uruguay. The following keywords were used in combinations in the database searches: ethnobiology, ethnoecology, ethnozoology, ethnopharmacology, ethnobiological, ethnobotanical, ethnobotany, ethnopharmacological, ethnoveterinary, and ethnoecological*.* These keywords include research in which the authors of these studies are assumed to be included in one of these fields.

Accessing all the published studies was not possible because some journals do not provide online access, and other journals restrict content. Therefore, our survey was limited to the most recent studies published between 1963 and 2012 (first semester). The search only included studies that directly investigated the relationship between human groups and different types of resources. Therefore, literature reviews and studies that presented purely pharmacological, phytochemical, or bromatological data were excluded. Studies whose content could be extracted from the abstracts and from additional information on the journal’s website were also included. We are aware that our selection criteria precluded the inclusion of many studies published in local journals and languages, but we preferred to use the broader databases to include international publications.

To compile the database, the following information was extracted after the paper selection:

a) Country of the first author’s affiliation: the affiliation information indicated in the paper was recorded. The nationality of the first author was initially examined, but this information was not available in the studies. Therefore, it was not possible to analyze this aspect;

b) Country in which the study was conducted: the location of the study described in the papers was considered. A paper could include studies located in more than one Latin American country;

c) Year of publication: as indicated in the journal;

d) Area of ethnobiological knowledge: works were classified primarily according to the classification of the authors of the study, categorized according to title, keywords, and a detailed reading of the text. The following areas of knowledge were considered: ethnoecology, ethnobiology, ethnobotany, ethnozoology, ethnopharmacology, ethnomedicine, ethnoveterinary and ethnomycology;

e) Number of study citations: number of study citations as indicated in the Scopus database. This number was used to verify that works with international collaboration are cited more often than those without international collaboration;

f) International collaboration: collaboration was recorded if the first author of the study was affiliated with an institution in the study country (even if s/he was from another country) and one or more of the secondary authors was affiliated with a foreign institution. We interpret the concept of international collaboration as the initiative of the Latin American researcher. If the survey was conducted on the initiative of a foreign researcher as part of individual projects, we do not consider this international collaboration. 

The goodness-of-fit G-test was used to verify whether significant differences were present in the number of published studies and international collaborations. The Chi-square test was used to assess significant differences in the proportion of studies published in each area of knowledge and the number of citations these papers received. The Chi-square test was also used to verify whether significant differences existed in the number of paper citations and international collaboration. Statistical analyses were performed using the software Bioestat 5.0 [12].

## Results and discussion

### How many studies were published per country and per area of knowledge, and how has this number varied over time?

**Figure 1:**
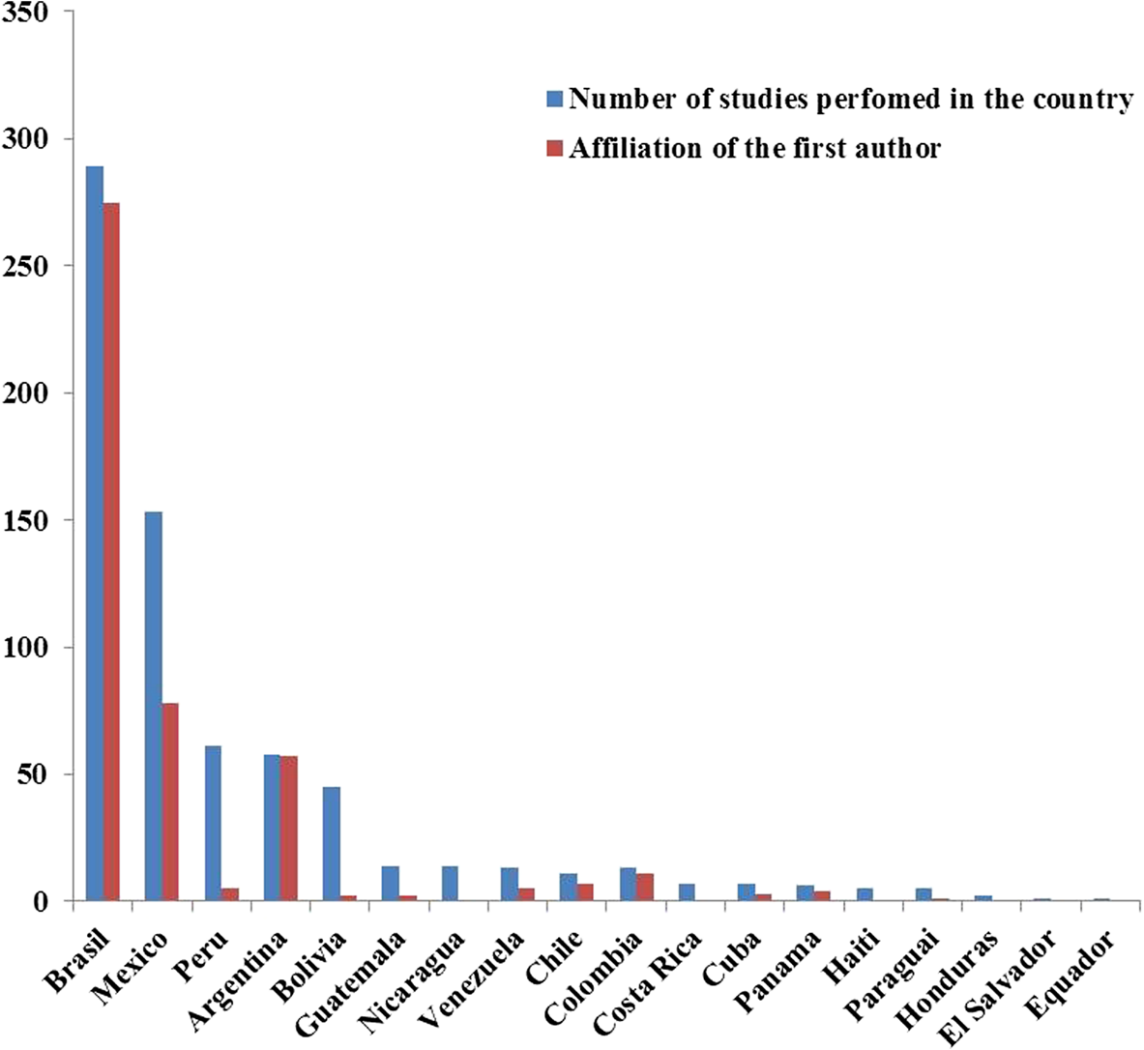
Number de ethnobiological studies in Latin America that were published between 1963 and 2012 and are available on the Scopus and Web of Science databases and affiliation of the first author.

A total of 679 ethnobiological studies performed in Latin America were identified for the period between 1963 and 2012. Of these studies, 289 (41%) were performed in Brazil, 153 in Mexico (22%), 61 in Peru (9%), 58 in Argentina (8%), 45 in Bolivia (6%), and 97 (14%) in the other Latin American countries (Figure 1). Importantly, Argentina, Brazil, and Colombia displayed autonomy in publishing studies; 98%, 95%, and 84% of the studies, respectively, were performed by researchers affiliated with institutions within these countries. Conversely, these numbers drop to 51%, 8%, and 4% in Mexico, Peru, and Bolivia, respectively (Figure 1). This lack of autonomy is even more conspicuous in Nicaragua and Costa Rica; none of the 14 and 7 studies, respectively, published in these countries was conducted by authors affiliated with institutions within these countries. The lack of “autonomy” in Peru, Bolivia, Nicaragua, Costa Rica may be a result, for example, of the lack of local research institutions promoting ethnobiology as a discipline or university courses.

Brazil was the Latin American country with the most published studies and the most first authors affiliated with institutions within the country. This result may reflect the increase in human resource training in ethnobiology in Brazil over the years in addition to publication of the first edition of the Brazilian Ethnobiological Compilation (Suma Etnológica Brasileira) [13], the First International Meeting of Ethnobiology (1988) held in Belem, North of Brazil (during which the International Society of Ethnobiology (ISE) was founded), and the foundation of the Brazilian Society of Ethnobiology and Ethnoecology (SBEE) in 1996 [11]. In addition, initiatives such as the creation of the first Brazilian publisher in ethnobiology and ethnoecology (NUPEEA) have strengthened and expanded professional, undergraduate, and graduate discussions on ethnobiology in the country [5].

Brazil also had the greatest number of papers in the areas of ethnobotany, ethnoecology, and ethnozoology (174, 30, and 26, respectively) (Table 1). An important clarification is that some papers had study areas located in more than one country; consequently, this table considered all of the papers from each country, for a total of 703 studies. The number of studies, especially in the area of ethnobotany, has grown exponentially (see [14]). Importantly, 101 of the ethnobotany studies were on medicinal plants. This high number of studies in medicinal plants has previously been reported by Oliveira et al. [11]. The remaining 73 ethnobotany studies addressed issues from general ethnobotany to more specific investigations, including the domestication and origin of agriculture, archaeobotany, agroforestry systems and backyards, forest uses, cognitive and historical studies, and research on fairs and public markets (see [15]). Regarding the ethnozoological studies, Alves and Souto [7] have indicated that the cultural and faunistic diversity of Brazil and the addition of ethnozoology to graduate programs may have contributed to an increase in scientific productivity in this area.

**Table 1:** Number (Nº) of papers and citations for the areas of ethnobiology in Latin American countries

**Countries**	**Area of ethnobiology**																	
	**Ethnobiology**		**Ethnobotany**		**Ethnoecology**		**Ethnopharmacology**		**Ethnomedicine**		**Ethnomycology**		**Ethnoveterinary**		**Ethnozoology**		**Total**	
	**Nº of papers**	**Nº of citations**	**Nº of papers**	**Nº of citations**	**Nº of papers**	**Nº of citations**	**Nº of papers**	**Nº of citations**	**Nº of papers**	**Nº of citations**	**Nº of papers**	**Nº of citations**	**Nº of papers**	**Nº of citations**	**Nº of papers**	**Nº of citations**	**Nº of papers**	**Nº of citations**
**Argentina**	0	0	53	331	1	3	1	5	3	37	0	0	0	0	0	0	58	376
**Bolivia**	2	6	31	333	2	29	3	59	7	44	0	0	0	0	0	0	45	471
**Brazil**	15	112	173	1885	30	160	23	136	17	283	0	0	5	32	26	393	289	3001
**Chile**	1	0	8	58	0	0	1	3	0	0	0	0	0	0	1	0	11	61
**Colombia**	0	0	9	168	0	0	2	31	0	0	0	0	0	0	0	0	11	199
**Costa Rica**	0	0	7	124	0	0	0	0	0	0	0	0	0	0	0	0	7	124
**Cuba**	0	0	7	78	0	0	0	0	0	0	0	0	0	0	0	0	7	78
**El Salvador**	0	0	1	3	0	0	0	0	0	0	0	0	0	0	0	0	1	3
**Ecuador**	0	0	1	3	0	0	0	0	0	0	0	0	0	0	0	0	1	3
**Guatemala**	0	0	10	125	2	34	1	6	1	0	0	0	0	0	0	0	14	165
**Haiti**	0	0	2	5	0	0	1	7	2	1	0	0	0	0	0	0	5	13
**Honduras**	0	0	2	19	0	0	0	0	0	0	0	0	0	0	0	0	2	19
**Mexico**	1	4	74	926	28	336	14	440	29	310	6	36	0	0	1	0	153	2052
**Nicaragua**	1	0	8	121	1	2	1	14	3	13	0	0	0	0	0	0	14	150
**Panama**	0	0	6	104	0	0	0	0	0	0	0	0	0	0	0	0	6	104
**Paraguay**	0	0	4	11	0	0	1	31	0	0	0	0	0	0	0	0	5	42
**Peru**	5	36	35	583	0	0	3	46	17	181	0	0	1	5	0	0	61	851
**Venezuela**	2	8	5	39	1	0	5	21	0	0	0	0	0	0	0	0	13	68

The lower number of publications in the area of ethnozoology compared to ethnobotany may be related to problems associated with the use of wild animals. Hunting is prohibited in Brazil, which hampers access to ethnozoology-related information because people who sell or use animal products avoid sharing this knowledge, impairing cooperation with researchers [7]. Notably, some studies that addressed the use of animals were considered to belong to other areas of knowledge based on the criteria adopted in the present analysis. For example, studies on the use and commerce of medicinal animals were included in the ethnomedicine category according to the way the work in this area was classified by the study authors. However, when we reclassified these works by placing them in ethnozoology, the identified relationships did not change. Therefore, the number of studies that used an ethnozoological approach was larger than 26, but still lower than ethnobotanical studies.

Mexico also stands out for its number of publications. Like Brazil, Mexico is considered to have a substantial number of publications [15]. In addition, Mexico was the only country with studies in the area of ethnomycology (6 papers). However, the first authors of fewer than half of the publications were affiliated with institutions within Mexico, which may indicate a gap in human resource training in this country. The increase in the number of studies in Mexico was stimulated by the establishment of the Mexican Association of Ethnobiology in 1993. Therefore, one of the challenges in strengthening ethnobiology in Mexico is investment in human resources training, as reported for Brazil.

Peru is another country with a substantial number of studies. However, many of these studies did not involve a first author affiliated with an institution within Peru, as mentioned above. The fact that foreign researchers travel to Latin America to conduct their research has been noted by Fonseca-Kruel et al. [16], who have shown that 52% of the publications in the area of ethnobotany in international journals were conducted in Latin America by North American, British, and French researchers.

In Argentina, the number of studies performed in the country and the number of studies whose first author was affiliated with an institution within Argentina was the same. This result is most likely due to the existence of research groups that specialize in ethnobiology. These groups are important for the consolidation of this field of investigation because the groups may perform long-term research and create standard studies that can subsequently be tested in different regions of the world.

The remaining Latin American countries did not have a substantial number of studies. This lack of publications suggests a deficiency in both the numbers of people specializing in ethnobiological research and the frequency of events, societies, and/or associations that boost the development of this research field in these countries. This finding demonstrates that in addition to the lack of people specializing in research and ethnobiological events and societies and/or associations that leverage the development of this area, other factors may influence the number of publications in these countries, such as the quality of training scientific research, the level of knowledge of English, access to good quality publications, funding for research, the strength of the research institutions, and the competitiveness of the academic work environment.

**Figure 2:**
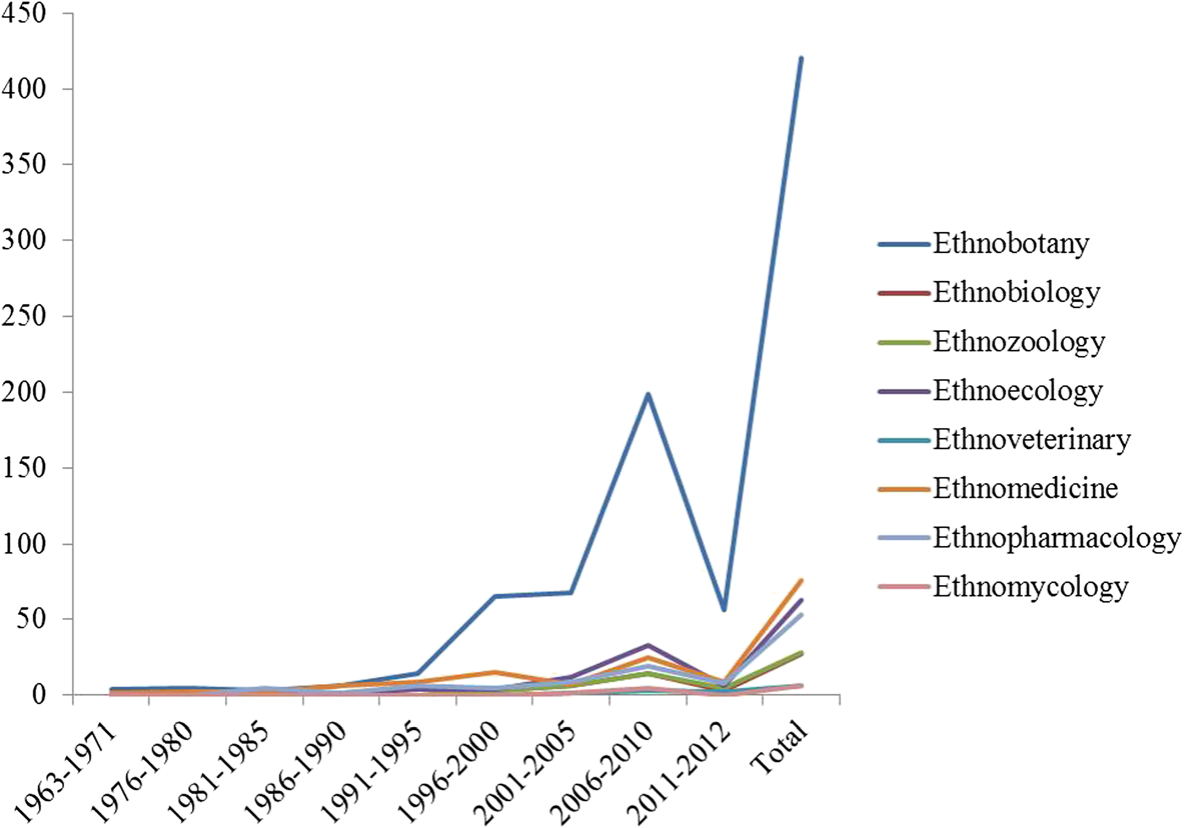
Number of papers published in Latin America between 1963 and 2012 in the different subareas of ethnobiology.

Among the ethnobiological areas of study that had the most publications over the years, special attention may be given to ethnobotany (420 papers), followed by ethnomedicine (76 papers), ethnoecology (63 papers), ethnopharmacology (53 papers), ethnozoology (28 papers), ethnobiology (27 papers), ethnoveterinary medicine (6 papers), and ethnomycology (6 papers) (Figure 2).

An increase was observed in the number of papers published between 1996 and 2012. These papers were primarily in the field of ethnobotany. The number of studies published between 2000 and 2005 did not change (Figure 2). During 2011 and 2012, 89 ethnobiology papers were published. Among these ethnobiology papers, 56 were ethnobotany papers, and 47 were papers from Brazil.

The number of ethnobotanical studies reflects the increase in the training of human resources in Brazil in recent years. For example, Brazilian universities offer graduate programs with research lines related not only to ethnobotany but also to other areas. In 2012, the Graduate Program in Ethnobiology and Conservation of Nature (PhD level) was created. This was the first graduate program in Latin America and launched the first Brazilian periodical, *Ethnobiology and Conservation*. The first ethnobiologists, who originally came from other specialties, were trained around the year 2000 by pioneer researchers in the field [16]. These researchers are currently training others in ethnobotany. Another important aspect is that, disciplines such as ethnomycology have appeared only recently, in comparison with ethnobotany. Therefore, the relatively low number of studies in this area compared to other areas of ethnobiology is not surprising.

### Do variations exist in the number of citations and international collaborations with regard to the number of publications by country and by area of knowledge?

**Figure 3:**
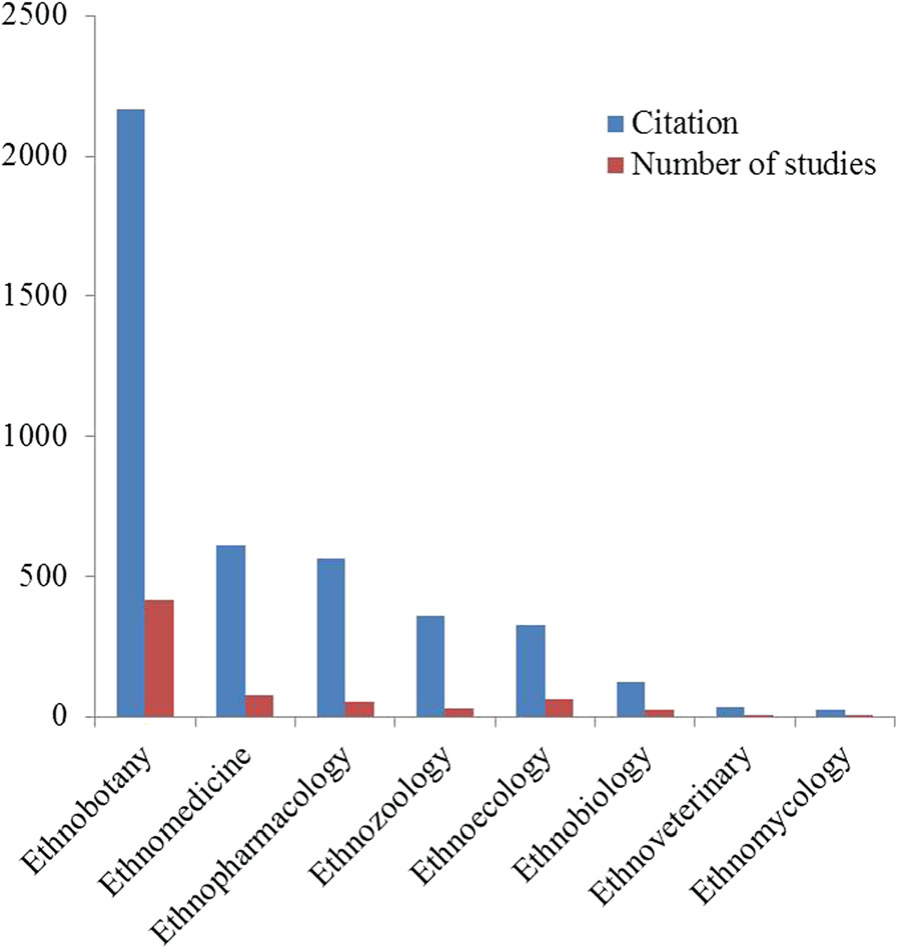
Total number of papers published in each area of knowledge available in the Scopus and Web of Science databases between 1963 and 2012 and number of citations for each area of knowledge.

Many factors may influence the number of citations these papers receive. The number of authors, names, gender, language, and interdisciplinary collaboration are some examples [17]. According to Abt [18], the duration of the research and the area of knowledge may also influence the number of citations received by scientific studies. Velho [19] has stated that when a study area is new, few studies can be cited, and citations concentrate on studies conducted in more recent areas of knowledge. When assessing the effect of the area of knowledge on the number of citations received by the articles included in our database (Figure 3), we observed that the areas with the most publications (ethnobotany, 420 papers, and ethnomedicine, 76 papers) have also received the most citations (2,168 and 612, respectively). The difference in the proportion of the number of citations relative to the number of published studies in the different areas was highly significant (*x*^2^ = 50.02, p < 0.0001).

The increased number of citations received by the papers in these first two areas may be explained by the fact that these are old areas in the ethnoscience field (the two oldest papers obtained in our study were published in 1963 and 1966 and belonged to the areas of ethnomedicine and ethnobotany, respectively). It is likely that a larger number of researchers work in these areas, leading to more studies and more citations. More studies were published in ethnoecology (63 papers) than in ethnopharmacology (53 papers), but the papers in ethnopharmacology have received more citations (563 citations) than those in ethnoecology (329 citations). The areas of ethnozoology and ethnobiology had a similar number of publications (28 and 27 papers, respectively), but the numbers of citations received by these two areas was quite different; ethnozoology received almost three times more citations (361) than ethnobiology (124). This difference highlights the importance and increased visibility of the ethnozoology field.

In the current study, the location in which the study was performed also influenced the number of citations. For example, studies performed in countries such as El Salvador and Ecuador had few citations (only three each, concentrated in the area of ethnobotany) (Table 1). Conversely, the number of citations was much higher in countries in which papers can easily be accessed by search engines (journals are indexed) and the COMUT library system (such as Brazil and Mexico, with 3,001 and 2,052 citations, respectively) (Table 1).

In the survey of studies that involved international collaboration, we identified studies that had a first author affiliated with an institution in the same country in which the research was performed and that included one or more authors from other countries. Of the total number of papers, we verified that only 35 studies involved international collaborations (receiving a total of 460 citations), and 644 studies did not involve international collaboration (receiving a total of 3,394 citations). However, studies that involved international collaboration received many more citations than those without collaboration (*x*^2^ = 27.289, p < 0.001). Therefore, we confirmed that international collaboration may contribute to an increased number of citations for a scientific study, most likely suggesting that studies with international collaboration have greater visibility and therefore receive more citations. Leimu and Koricheva [20] have stated that scientific collaboration is often associated with the quality of the science. These authors have verified that studies with interdisciplinary collaborations between different institutions receive more citations than do those without collaboration.

International collaborations were found in each of the following countries: Brazil (11 papers), Mexico (10 papers), Peru (4 papers), Bolivia (2 papers), and Venezuela (3 papers), followed by Cuba, Colombia, Argentina, Costa Rica, Panama, and Honduras (1 paper each) (Table 2). No international collaborations were identified among the studies performed in Guatemala, Nicaragua, Chile, Haiti, Paraguay, El Salvador, and Ecuador. The goodness-of-fit G-test for significant differences between the number of published studies and the respective number of international collaborations among the countries with the most publications (Brazil, Mexico, and Peru) indicated no significant difference between Brazil and Mexico (G-test = 02.32; p = 0.8272), Brazil and Peru (G-test = 2.1656; p = 0.0653), or Mexico and Peru (G-test = 1.3608; p = 0.1031).

**Table 2:** Proportion of papers published in collaboration with international researchers in the different countries

**Country**	**Number of papers**	**Number of papers with international collaboration**	**International collaboration (%)**
Brazil	289	11	4
Mexico	153	10	7
Peru	61	4	7
Argentina	58	1	2
Bolivia	45	2	4
Guatemala	14	0	0
Nicaragua	14	0	0
Venezuela	13	3	23
Chile	11	0	0
Colombia	11	1	9
Costa Rica	7	0	0
Cuba	7	1	14
Panama	6	1	17
Haiti	5	0	0
Paraguay	5	0	0
Honduras	2	1	50
El Salvador	1	0	0
Ecuador	1	0	0

The considerable number of international collaborations in Brazil and Mexico, which was greater than that in the other countries, may be related to the megadiversity found in these countries, the fact that these countries have the largest number of published papers, or the expressive insertion situation in the international scientific community. In studies of Latin American biodiversity, nations tend to seek partners with technical-scientific knowledge and financial resources (usually from North America) to aid in the exploration of their biodiversity [21].

However, the investigation of the proportion of studies with international collaboration and the total number of studies indicated that the countries with more published studies tended to have a low percentage of studies that were developed in collaboration with international researchers (Table 2). A higher number of publications in a given area may reflect diffusion and consolidation of the field, which may motivate the development of human resources and thus reduce the need for partnership with researchers from international institutions.

The higher proportion of international collaborations in countries with fewer publications may be associated with the role these collaborations play in strengthening research areas in the country. This association between the development of science and the interactions between different scientists has been noted by Vanz and Stump [22], who consider these interactions important steps toward the advancement and establishment of certain areas of knowledge. These interactions between researchers may facilitate the development of large research projects in a shorter time span and with less effort, thus increasing the productivity and quality of the research [23].

With regard to the areas of ethnobiology, a higher number of papers with scientific collaboration was recorded for the area of ethnobotany (21 papers), followed by ethnopharmacology (4 papers), ethnobiology (3 papers), ethnoecology, ethnomedicine, and ethnoveterinary medicine (2 papers each), and ethnozoology (1 paper). No international collaboration was observed in the area of ethnomycology.

Oliveira et al. [11] have highlighted the important role of foreign researchers in ethnobotanical studies performed in Latin America. However, investigation of the proportion of international collaborations in the studies published in different areas of knowledge indicate that international partnership was involved in only 5% of the ethnobotanical studies (Table 3). This percentage of international collaboration among ethnobotany studies is lower than that in other areas, such as ethnoveterinary medicine, ethnobiology, and ethnopharmacology.

**Table 3:** Proportion of papers published in collaboration with international researchers in each area of knowledge

**Area of knowledge**	**Number of papers**	**International collaboration**	**International collaboration (%)**
Ethnobotany	399	21	5
Ethnomedicine	74	2	3
Ethnoecology	61	2	3
Ethnopharmacology	49	4	8
Ethnozoology	27	1	4
Ethnobiology	24	3	13
Ethnomycology	6	0	0
Ethnoveterinary	4	2	50

The high proportion of international collaborations observed for ethnoveterinary medicine and ethnopharmacology may be related to interest in the products that can be generated as a result of these studies. Fonseca-Kruel et al. [16] have stated that new technologies, methods, and molecular characterizations of biological resources stimulate interest in the search for new commercial products. In addition, developed countries have a strong interest not only in ethnopharmacological studies but also in the local and traditional knowledge that can be found in developing countries [21].

## Conclusions

Notwithstanding the notorious growth of ethnobiology as a science in Latin America (as evidenced by the increase in ethnobiology publications over the years), the number of publications seems to lag in many countries. Overall, our findings on the development of ethnobiology in Latin America, including Brazil, Mexico, and Argentina, reproduce large-scale occurrences in the development of scientific research in the region, as demonstrated by the SCOPUS database (http://www.scopus.com). Huggett [24] used this database to produce a very interesting article on the growth of scientific research in Latin America.

This situation could be altered by the incorporation of greater incentives for ethnobiology research through investment in human and financial resources and the creation of research groups and ethnobiological societies (as observed in Brazil and Mexico). These groups may promote and expand discussions on the subject, create protocols, and generate knowledge that may fill the existing gaps in many areas of ethnobiology (see [25]). Ethnobiological research may also be stimulated by an increase in the number of scientific events and journals to disseminate studies and by the creation of undergraduate courses and graduate programs to train ethnoscientists who will produce high-quality studies. These approaches can address the gaps and lags in ethnobiological research, contributing to the consolidation of ethnobiological knowledge throughout Latin America, as has been observed in ethnobiological research in countries such as Brazil, Mexico, Peru, and Argentina.

## Competing interests

The authors declare that they have no competing interests.

## Authors’ contributions

UPA, JSS, JLAC, RSS, TCS, RRNA – Writing of the manuscript, literature survey and interpretation, and data analysis. All authors read and approved the final manuscript.
